# First-Generation and Low-Income Students in the National Medical Student Body

**DOI:** 10.1001/jamanetworkopen.2025.9769

**Published:** 2025-05-12

**Authors:** Sophia C. Kamran, Isabella R. Pompa, Hillary Brenda Nguyen, Jaeyoon Cha, Kevin E. Salinas, Andrzej Niemierko, Neha Vapiwala

**Affiliations:** 1 Department of Radiation Oncology, Massachusetts General Hospital, Boston; 2 Harvard Medical School, Boston, Massachusetts; 3 Transitional Year Program, John Peter Smith Hospital, Fort Worth, Texas; 4 Department of Radiation Oncology, Hospital of the University of Pennsylvania, Perelman School of Medicine, Philadelphia

## Abstract

**Importance:**

Diverse representation within the US medical student body and physician workforce is an important step to help address known pervasive health care disparities.

**Objective:**

To evaluate US medical school first-year matriculants between 2002 and 2015 through a disaggregated lens, parsing out low-income and first-generation students and understanding their intersectionality with racial and ethnic groups.

**Design, Setting, and Participants:**

Using deidentified student-level data of allopathic US medical school matriculants from the Association of American Medical Colleges Matriculating Student Questionnaire from 2002 through 2015, this cross-sectional study obtained demographic data with a focus on first-generation and low-income status. Data were analyzed from January 2022 through July 2024.

**Exposures:**

Race and ethnicity, first-generation medical student status, underrepresented in medicine (URIM) status (ie, identifying as Hispanic only, non-Hispanic American Indian or Alaska Native or Native Hawaiian or Pacific Islander only, or non-Hispanic Black/African American only), parental income, total student debt, and graduation status.

**Main Outcomes and Measures:**

Trends of first-generation and low-income students were evaluated longitudinally. Medical student parental income was compared with that of the US general population by race and ethnicity and generation status for the years 2002, 2008, and 2015. A multivariable logistic regression model was performed to evaluate URIM, parental income, first-generation status, and attrition from medical school.

**Results:**

Between 2002 and 2015, a total of 256 513 students were included in this analysis (48.0% women; 14.7% URIM; 7.4% first-generation). The proportion of first-generation students decreased from 8.7% in 2002 to 7.1% in 2015. When comparing student parental income with that of the general population for 2002, 2008, and 2015, the top 5% of households by income were overrepresented among non–first-generation medical students (21.1% in 2002, 31.5% in 2008, and 26.4% in 2015), while the parental income representation of first-generation students was more aligned with the general population (1.4% in 2002, 2.8% in 2008, and 0.8% in 2015). The graduation rate of first-generation students was 2.1% lower than the graduation rate of non–first-generation students. Medical students were more likely to not graduate if they were low-income (odds ratio [OR], 1.71 [95% CI, 1.60-1.84]), URIM (OR, 2.07 [95% CI, 1.98-2.17]), or first-generation (OR, 1.56 [95% CI, 1.47-1.67]).

**Conclusions and Relevance:**

This cross-sectional study of US medical student matriculants found a decrease in the number of matriculants who were first-generation. These students were at significant risk of attrition from medical school, particularly when considering the intersectionality with low-income and URIM identities. These results suggest a need to recruit and retain these students, so that the physician workforce better reflects the backgrounds and experiences of the communities served.

## Key Points

**Question:**

Did the diversity of US medical school matriculants evolve with respect to first-generation and low-income status between 2002 and 2015?

**Findings:**

In this cross-sectional study involving 256 513 US medical school students, first-generation matriculants decreased over time, while the proportion of students with parental income in the top 5% of US households increased. Furthermore, the attrition rate of first-generation students was substantial, particularly when evaluating the intersectionality of parental income, parental educational level, and underrepresented race and ethnicity.

**Meaning:**

These findings suggest that policies to recruit and retain socioeconomically diverse individuals are needed to improve both the educational environment for future physicians and health care for all in the US.

## Teaser

This cross-sectional study assesses whether parental income, underrepresented race and ethnicity in medicine, and attrition rates changed from 2002 to 2015 among first-generation and non–first-generation US medical school first-year matriculants.

## Introduction

Despite decades of medical breakthroughs and advances, health disparities remain pervasive among underserved communities. Diverse representation within the US medical student body is an important step to help address these disparities, with proven benefits including improved quality of care, increased innovation, stronger teamwork, and decreased implicit biases toward patients.^1,2,3^ Heightened focus on gender, racial, and ethnic diversity among qualified applicants has yielded some improvement over the years that nonetheless falls short of reflecting the general US population.^2^

Socioeconomic representation within medicine has only recently received some attention, including first-generation students (defined as individuals whose parents did not complete a baccalaureate degree, per the Higher Education Act of 1965 and 1998).^4^ While socioeconomic diversity and first-generation status can be highly intersectional with individuals from racial and ethnic groups underrepresented in medicine (URIM), they transcend racial and ethnic boundaries and represent a unique, more expansive group.^5^ Socioeconomic imbalance among US medical students has been previously characterized, demonstrating little to no improvement over the past few decades.^6^ However, data have never been previously disaggregated, and thus comparatively little is known about first-generation students. Herein, we present a large, longitudinal, comprehensive analysis of first-generation medical students within the US national medical student body, to gain insight into first-generation diversity trends in the physician workforce.

## Methods

### Database

Deidentified student-level data of allopathic US medical school first-year matriculants were acquired for this cross-sectional study from the Association of American Medical Colleges (AAMC) Matriculating Student Questionnaire (MSQ)^7^ administered annually to all first-year enrollees from 2002 to 2015 and corresponding Graduating Student Questionnaire (GSQ)^8^ from 2005 to 2020. The MSQ has undergone 3 major revisions (1997, 2007, and 2013) and has a response rate between 66% and 77%, while the GSQ has a response rate of about 80%.^9^ Our analysis focused on race and ethnicity, first-generation status, URIM status,^10^ parental income, total debt, and graduation status. This study was exempted from human participant research guidelines and the need for informed consent by the Massachusetts General Brigham Institutional Review Board due to its being a secondary analysis of existing deidentified data. The study followed the Strengthening the Reporting of Observational Studies in Epidemiology (STROBE) reporting guideline.

Based on the AAMC First-Generation-College-Student Indicator definition, students were identified as first-generation if they selected that the educational levels of both parents were less than high school, high school graduate (high school diploma or equivalent), or some college but no degree.^11^ As this indicator was not put into use until 2017, students were manually classified based on their MSQ responses. Students listing only 1 parent were classified as first-generation if the parent had an educational level of less than high school, high school graduate (high school diploma or equivalent), or some college but no degree. Students were identified as low-income if their parental income fell in the bottom 2 quintiles based on the US census household income quintiles for the year that they completed the MSQ.^12^ Adjusting parental income for inflation was unnecessary as US census quintile boundaries change annually (eTable 1 in Supplement 1). Total parental income was separated into quartiles, by year, among the entire student population to evaluate the proportion of students falling into each quartile. Total educational debt at graduation was binned into quartiles for each graduating class annually with changing boundaries; therefore, adjustment for inflation was also not needed (eTable 2 in Supplement 1). For each race and ethnicity, medical student household income was compared with that of the general population in 2002, 2008, and 2015,^13^ selected as representative years over the study duration. Race and ethnicity were self-reported by students and categorized as Hispanic; non-Hispanic American Indian or Alaska Native or Native Hawaiian or Pacific Islander; non-Hispanic Asian (Asian); non-Hispanic Black (Black); non-Hispanic White (White); non-Hispanic multiracial; and other, unknown, or declined. Students reporting 2 or more races were categorized as multiracial. To standardize race classifications for surveys between 2002 and 2012 and between 2013 and 2015, for the former, we classified Asian Indian, Chinese, Filipino, Japanese, Korean, Pakistani, Vietnamese, and other Asian as Asian, and American Indian or Alaska Native, Guamanian or Chamorro, Native Hawaiian, Samoan, and Other Pacific Islander as non-Hispanic American Indian or Alaska Native or Native Hawaiian or Pacific Islander. We also classified Chicano/Chicana, Cuban, Mexican, Mexican American, Puerto Rican, and other Hispanic as Hispanic for the years 2002 to 2012. URIM referred to students who identified as Hispanic only; non-Hispanic American Indian or Alaska Native or Native Hawaiian or Pacific Islander only; or non-Hispanic Black or African American only, per the AAMC definition.^9^

To evaluate attrition from medical school, we examined the matched GSQ for first-year matriculants enrolled between 2002 and 2012 to determine whether they graduated successfully, allowing for at least 8 years of follow-up for all students to account for MD-PhD program candidates.

### Statistical Analysis

For parental income, medical students were categorized based on US census household income quintiles. Simple linear regression models were used to estimate trends and associated *P* values, with year as the independent variable. For each race and ethnicity, medical student parental income was compared with that of the general population, by generation status (first vs not first). We defined a representation index (RI) for each subgroup, based on the ratio of the proportion of the medical student subgroup vs the general US population. Values of 1.0 or above indicated adequate or overrepresentation in the medical student subgroup; values below 1.0 indicated underrepresentation in the medical student subgroup. To evaluate categories of students graduating vs not graduating, χ^2^ analyses were performed. A 2-sided *P* < .05 with multiple hypothesis test correction by the Holm-Bonferroni method was considered statistically significant. Separately, a multivariable logistic regression model was performed to understand the association between URIM, parental income, first-generation status, and attrition from medical school. A 2-sided *P* < .05 indicated statistical significance. All statistical analyses were performed from January 2022 to July 2024 using R, version 4.3.0 (R Project for Statistical Computing).

## Results

**Figure 1.**
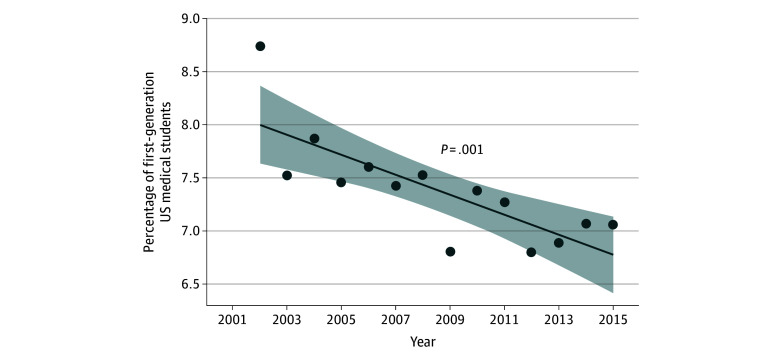
Percentage of First-Generation US Medical Students Within US Medical Student Body The data are presented as a percentage of the total by Matriculating Student Questionnaire year, 2002 through 2015. The line represents the linear regression fit; the dots represent the individual data points for each year (ie, the percentage of first-generation students in the medical student population per year); and the shaded area represents the 95% CI.

Between 2002 and 2015, a total of 256 513 students were included in the analysis. Of these students, 123 088 (48.0%) were women and 133 421 (52.0%) were men. By race and ethnicity, 20 517 students (8.0%) identified as Hispanic; 997 (0.4%) as non-Hispanic American Indian or Alaska Native or Native Hawaiian or Pacific Islander; 50 029 (19.5%) as non-Hispanic Asian; 16 263 (6.3%) as non-Hispanic Black; 147 607 (57.5%) as non-Hispanic White; 8069 (3.1%) as non-Hispanic multiracial; and 13 031 (5.1%) as other or unknown (eTable 3 in Supplement 1). In total, 37 777 students (14.7%) were URIM. Of 242 059 students with parental educational data, 17 807 (7.4%) identified as first-generation. Over time, the proportion of first-generation students steadily decreased, from 8.7% in 2002 to 7.1% in 2015 (*P* = .001) (Figure 1). Among 158 127 students with parental income data, 16 978 (10.7%) were identified as low-income.

### Parental Income per US Census Household Income Quintiles per Year

**Figure 2.**
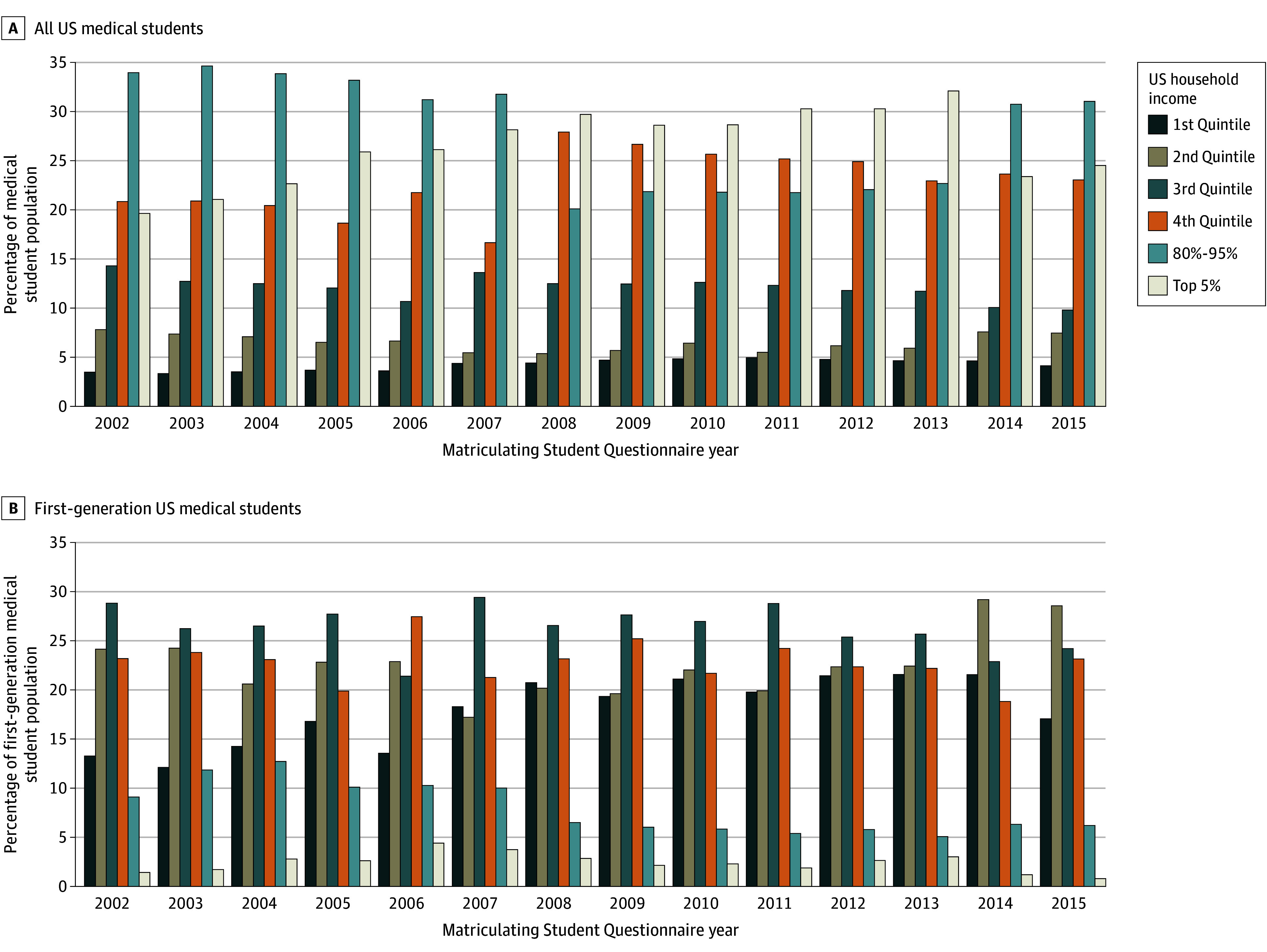
Parental Income of US Medical Student Matriculants by Quintiles of US Household Income

Between 2002 and 2015, the proportion of medical students with parental income in the top 5% of US household income increased over time (from 19.6% in 2002 to 24.5% in 2015), as did the proportions with parental income in the highest (fourth) quintile (20.8% in 2002 to 23.0% in 2015) (Figure 2A; eTable 4 in Supplement 1). When examining first-generation medical students separately, the proportion of students with parental income in the top 5% of US household income was very small (<5% of all first-generation students) and stagnant over time (from 1.4% in 2002 to 0.8% in 2015), while the proportion of first-generation students with parental income in the lowest (first) quintile increased over time (from 13.3% in 2002 to 17.1% in 2015) (Figure 2B, eTable 4 in Supplement 1). When comparing proportions of first-generation and non–first-generation US medical students with parental income in the top 5% of US household incomes, year-by-year, there was a higher proportion of non–first-generation medical students in this category compared with first-generation medical students (21.1% in 2002, 31.5% in 2008, and 26.4% in 2015 for non–first-generation vs 1.4% in 2002, 2.8% in 2008, and 0.8% in 2015 for first-generation) (eTable 4 in Supplement 1). Conversely, when comparing proportions of first-generation and non–first-generation medical students with parental income in the lowest (first) quintile of US household incomes, year-by-year, these were different, with a higher proportion of first-generation medical students in this category compared with non–first-generation medical students (eg, 3.3% non–first-generation vs 20.7% first-generation in 2008) (eTable 4 in Supplement 1).

### Parental Income Among Total Medical Student Population per Year

The percentage of first-generation students in the lowest quartile of parental income per year remained stable between 2002 (25.0%) and 2015 (22.3%) (*P* = .84) (eFigure 1A, eTable 3 in Supplement 1). When further examining the excess percentage of first-generation students in the lowest quartile of parental income per year, there was a significantly increasing number of first-generation students with lower-income parents per year (187.7% in 2002 to 215.6% in 2015, *P* = .006) (eFigure 1B, eTable 3 in Supplement 1). The median parental income among first-generation students remained stable over the years, while that of non–first-generation students increased per year; the ratio therefore decreased markedly over time, from 0.44 ($40 000/$90 000) in 2002 to 0.36 ($45 000/$125 000) in 2015 (*P* = .007) (eFigure 2, eTable 3 in Supplement 1).

### US Medical Student Educational Debt and Residency Selections

Graduating first-generation medical students had a higher amount of total educational debt compared with non–first-generation students, with a significantly increasing debt ratio between 2002 (ratio, 1.12) and 2015 (ratio, 1.31) (*P* = .004) (eFigure 3, eTable 3 in Supplement 1). The most common residency specialties pursued by graduates overall included internal medicine (25.8%), followed by surgery (14.0%) and pediatrics (11.9%). These were the top 3 residency specialties for non–first-generation students as well (26.0% for internal medicine, 14.1% for surgery, and 12.1% for pediatrics). Compared with first-generation graduates, a lower proportion of non–first-generation graduates entered family medicine (11.8% vs 8.1%), a higher proportion chose to pursue pediatrics (9.7% vs 12.1%) or surgery (12.8% vs 14.1%), and fewer students had a decision that was not documented at the time of graduation (10.9% vs 8.4%) (eTable 5 in Supplement 1).

### Intersectional Evaluation of Socioeconomic Status by First-Generation Status and Race and Ethnicity

**Figure 3.**
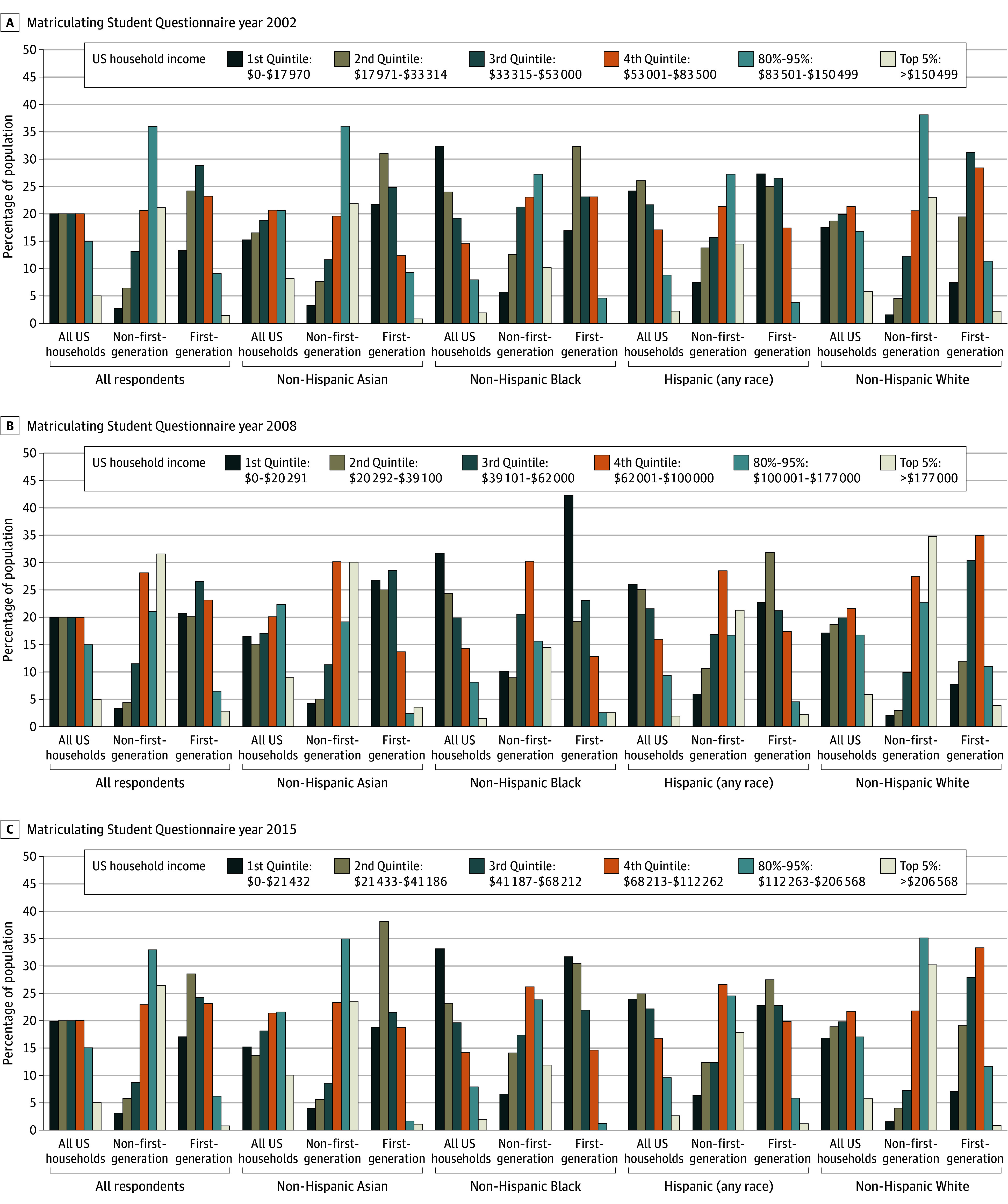
Comparison of Socioeconomic Composition of US Households, US Non–First-Generation Medical Students, and US First-Generation Medical Student Matriculants by Race and Ethnicity The non-Hispanic American Indian or Alaska Native or Native Hawaiian or Pacific Islander category was omitted as there were limited data from the Matriculating Student Questionnaire, and this category was not listed as a classification for all US Households in the US census.

A comparison of parental income composition of non–first-generation and first-generation medical students by each racial and ethnic group between the medical student body and the US population was performed for the years 2002, 2008, and 2015. In 2002, the top 5% of households by income were overrepresented, with RIs higher than 1.0 among non–first-generation medical students compared with the US population (RI, 4.2 [21.1% vs 5.0%] for all; RI, 2.7 [21.9% vs 8.1%] for Asian; RI, 5.3 [10.2% vs 1.9%] for Black; RI, 6.5 [14.5% vs 2.2%] for Hispanic; and RI, 4.0 [23.0% vs 5.8%] for White students) (Figure 3A, Table 1; eTable 6A in Supplement 1). The remainder of the top quintile (80%-95%) was overrepresented (RI ≥ 1.0) for each race and ethnicity among non–first-generation medical students. First-generation students were consistently underrepresented in the top quintile for each race and ethnicity, with no representation for first-generation Black or Hispanic students. First-generation Hispanic students were overrepresented in the lowest quintile compared with the US population (RI, 1.1 [27.3% vs 24.2%]). First-generation Asian students were overrepresented in the lowest quintile compared with the US population (RI, 1.4 [21.7% vs 15.2%]).

**Table 1.** Intersection of Socioeconomic Status and First-Generation Status by Race and Ethnicity

Category of household by income	Representation index for non–first-generation or first-generation students vs all US households												
	All students		Non-Hispanic Asian students			Non-Hispanic Black students			Hispanic (any race) students			Non-Hispanic White students	
	Non–first-generation	First-generation	Non–first-generation	First-generation	Non–first-generation		First-generation	Non–first-generation		First-generation	Non–first-generation		First-generation
**2002**													
Top 5%	4.2	0.3	2.7	0.1	5.3		0	6.5		0	4.0		0.4
80%-95%	2.3	0.6	1.7	0.5	3.4		0.6	3.1		0.4	2.3		0.7
Fourth quintile	1.0	1.2	0.9	0.6	1.6		1.6	1.3		1.0	1.0		1.3
Third quintile	0.7	1.4	0.6	1.3	1.1		1.2	0.7		1.2	0.6		1.6
Second quintile	0.3	1.2	0.5	1.9	0.5		1.3	0.5		1.0	0.2		1.0
First quintile	0.1	0.7	0.2	1.4	0.2		0.5	0.3		1.1	0.1		0.4
**2008**													
Top 5%	6.3	0.6	3.4	0.4	9.4		0	11.0		1.2	5.9		0.7
80%-95%	1.4	0.4	0.9	0.1	1.9		0.3	1.8		0.5	1.4		0.7
Fourth quintile	1.4	1.2	1.5	0.7	2.1		0.9	1.8		1.1	1.3		1.6
Third quintile	0.6	1.3	0.7	1.7	1.0		1.2	0.8		1.0	0.5		1.5
Second quintile	0.2	1.0	0.3	1.7	0.4		0.8	0.4		1.3	0.2		0.6
First quintile	0.2	1.0	0.3	1.6	0.3		1.3	0.2		0.9	0.1		0.5
**2015**													
Top 5%	5.3	0.2	2.3	0.1	6.3		0	6.8		0.4	5.3		0.1
80%-95%	2.2	0.4	1.6	0.1	3.0		0.2	2.6		0.6	2.1		0.7
Fourth quintile	1.2	1.2	1.1	0.9	1.8		1.0	1.6		1.2	1.0		1.5
Third quintile	0.4	1.2	0.5	1.2	0.9		1.1	0.6		1.1	0.4		1.4
Second quintile	0.3	1.4	0.4	2.8	0.6		1.3	0.5		1.1	0.2		1.0
First quintile	0.2	0.9	0.3	1.2	0.2		1.0	0.3		1.0	0.1		0.4

In 2008, the top 5% of households by income were again overrepresented among non–first-generation medical students compared with the US population, with higher RIs compared with 2002 (RI, 6.3 [31.5% vs 5.0%] for all; RI, 3.4 [30.1% vs 8.9%] for Asian; RI, 9.4 [14.5% vs 1.5%] for Black; RI, 11.0 [21.3% vs 1.9%] for Hispanic; and RI, 5.9 [34.8% vs 5.9%] for White students) (Figure 3B, Table 1; eTable 6B in Supplement 1). First-generation students were similarly underrepresented in the top 5%, with the exception of Hispanic first-generation students (RI, 1.2 [2.3% vs 1.9%]). The proportion of Black first-generation students in the lowest quintile was not much different than that of the US population (RI, 1.3 [42.3% vs 31.7%]).

Similarly, in 2015, the top 5% of households by income were consistently overrepresented among non–first-generation medical students compared with the US population, regardless of race or ethnicity (RI, 5.3 [26.5% vs 5.0%] for all; RI, 2.3 [23.5% vs 10.1%] for Asian; RI, 6.3 [11.9% vs 1.9%] for Black; RI, 6.8 [17.8% vs 2.6%] for Hispanic; and RI, 5.3 [30.2% vs 5.7%] for White students) (Figure 3C, Table 1; eTable 6C in Supplement 1). All first-generation students were underrepresented in the top 5% of households by income, with no representation of Black first-generation medical students. The bottom 3 quintiles were consistently underrepresented for non–first-generation medical students (RI < 1.0). There was no large difference between the proportion of first-generation medical students in the lowest quintile and that of the US population for all first-generation (RI, 0.9 [17.1% vs 19.9%]), Asian first-generation (RI, 1.2 [18.8% vs 15.2%]), Black first-generation (RI, 1.0 [31.7% vs 33.2%]), and Hispanic first-generation (RI, 1.0 [22.8% vs 24.0%]) students.

### Medical Student Attrition

There were 195 493 students with matched graduation status data. Of these, 6860 (3.5%) did not graduate, while 188 633 (96.5%) of students graduated (eTables 7 and 8 in Supplement 1). Of the 6860 students who did not graduate, 10.6% (725) were first-generation. This is in comparison to 6.8% first-generation students (12 932 of 188 633) who did graduate (*P* < .001). Additionally, 9.8% of nongraduating students (672 of 6860) were considered low-income vs 6.5% of low-income students who graduated (12 279 of 188 633) (*P* < .001). Over time, the graduation rate of first-generation students was approximately 2.1% lower than that of non–first-generation students (eg, non–first-generation graduation rate of 96.9% in 2002 and 95.9% in 2012 vs first-generation graduation rate of 94.5% in 2002 and 93.6% in 2012) (*P* < .001) (eTable 7, eFigure 4A, in Supplement 1). When considered using parental income quintiles based on all survey-reported incomes—as opposed to US-household income quintiles—students in the highest income group had a higher probability of graduation compared with students with parents in the lowest income group. This difference in probability of graduation between the highest income and lowest income groups was statistically significant, ranging between 2.2% and 2.9% (*P* < .001) (eFigure 4B in Supplement 1).

When evaluating medical students with complete data for known graduation status, low-income status, first-generation status, and race and ethnicity, which constituted a total of 115 015 medical students, 3538 students did not graduate (3.1%) (Table 2). Students had higher odds of not graduating if they were low-income (OR, 1.71 [95% CI, 1.60-1.84]), URIM (OR, 2.07 [95% CI, 1.98-2.17]), or first-generation (OR, 1.56 [95% CI, 1.47-1.67]). For students who identified as low-income, URIM, and first-generation, the OR for attrition was 2.51 (95% CI, 2.12-2.97).

**Table 2.** Intersections in Attrition From Medical School With Race and Ethnicity, Parental Income, and First-Generation Status, 2002 Through 2012

Variable	Students, total No.	Student attrition, No. (%)	Multivariable analysis, OR of attrition (95% CI)
All students^a^	115 015	3538 (3.1)	NA
Low-income^b^			
No	103 287	2948 (2.9)	1 [Reference]
Yes	11 728	590 (5.0)	1.71 (1.60-1.84)
URIM^c^			
No	99 168	2606 (2.6)	1 [Reference]
Yes	15 847	932 (5.9)	2.07 (1.98-2.17)
First-generation			
No	106 455	3098 (2.9)	1 [Reference]
Yes	8560	440 (5.1)	1.56 (1.47-1.67)

Abbreviations: OR, odds ratio; URIM, underrepresented in medicine.

^a^ Students were removed from analysis if data were unknown for these variables.

^b^ Low-income includes parental income in the bottom 2 quintiles for that year.

^c^ URIM included Hispanic; non-Hispanic American Indian or Alaska Native or Native Hawaiian or Pacific Islander; and non-Hispanic Black or African American.

## Discussion

As the US population continues to diversify, it is crucial that our health care system prioritizes an appropriately representative workforce.^14^ Efforts to promote diversity within medicine are applauded, yet to date have mostly focused on gender, race, and ethnicity. Socioeconomic diversity within medical schools has stagnated,^6^ and little is known about students who identify with first-generation status, which—given the powerful correlation between parental educational level and household income—is closely intertwined with low-income status.^15^ This demographic was only recognized by the AAMC in 2017^5^; thus, virtually no information exists surrounding these students and their trajectories. Greater access to medical training for students from lower socioeconomic backgrounds could improve overall quality of care and physician-patient relationships, as well as help to ensure sufficient health care professionals for an aging population.^16^

In this analysis of 256 513 US medical students between 2002 and 2015, we found that the proportion of first-generation first-year matriculants decreased over time, in contrast to relatively stable numbers of US first-generation undergraduate students previously reported between 2015 and 2016 (56%)^17^ and between 2019 and 2020 (54%).^18^ This is likely due to the observed decrease in first-generation applicants over time.^19^ We also found that the proportion of medical students with high parental income (top 5% of US households) significantly increased over time, while that of first-generation students with the lowest parental income (first quintile) also increased. Longitudinally, the graduation rate of first-generation students was approximately 2.1% lower than the graduation rate of non–first-generation students. Medical students were more likely to not graduate if they were low-income, URIM, or first-generation. For those who identified as having all 3 identities, the odds ratio for attrition was higher.

For decades, medical students have disproportionately come from high-income households,^20^ with little improvement in socioeconomic diversity.^6^ Our results are consistent with this finding, particularly among non–first-generation medical students, who are overrepresented for parental income in the top 5% of US households, regardless of race or ethnicity. On the other hand, first-generation medical students often come from the bottom quintiles of US household income,^21,22^ and in proportions consistent with those found throughout the US population by race and ethnicity. Therefore, these students are representative of the US general patient population that is seen throughout the health care system. First-generation students are an often overlooked and invisible minority among diversity discussions, although they bring a much-needed perspective to medicine.^23^ Many have had similar experiences to the patient population they serve and thus have cultural understanding, compassion, and empathy,^5,16^ creating a unique physician-patient relationship that can improve health care delivery and outcomes. In addition, it is well established that teams with diverse perspectives are more productive and creative,^24^ and health care teams that reflect the demographic characteristics of their patients lead to improved patient compliance, outcomes, and satisfaction.^25^ However, unique challenges persist in attracting and retaining this population within the medical field, which requires a multipronged approach and policies to foster their success throughout the training and early career pipeline and will aid in improving diversity among the physician workforce downstream.^5,16^

### Limitations

Our study is limited by the validity of self-reported parental income, parental educational level, graduation status, race and ethnicity, and student debt on the AAMC questionnaires, as these responses could be subject to bias. Nonrespondents or respondents with incomplete survey data were not included, which could affect the findings. In addition, the analysis was limited to a few demographic variables, there was no additional information on graduation degree, and we could not confirm whether individuals were in an MD-only program vs an MD-PhD program. However, this is, to our knowledge, the largest longitudinal analysis of first-generation medical students compared with non–first-generation medical students, demonstrating crucial findings that can aid in future recruitment of first-generation students and development of policies to ensure their success.

## Conclusions

In this cross-sectional study of US medical student first-year matriculants between 2002 and 2015, we report a decrease in the number who are the first in their families to graduate from college. These students were often classified as low-income, coming from the bottom quintiles of US household income. First-generation students were at significant risk of attrition from medical school, particularly when considering the intersectionality with low-income and URIM identities, which furthered their risk of attrition. This is one of the first reports to comprehensively evaluate US medical student demographic data by first-generation status. These results indicate a pressing need for intentional investment in recruiting and retaining these students, with the goal of diversifying the physician workforce to better reflect the communities we serve.

## Notes

Supplement 1. **eFigure 1.** Parental income of first-generation U.S. medical students **eFigure 2.** Ratio of median first-generation U.S. medical student parental income to non-first-generation U.S. medical student parental income, expressed as percent per year **eFigure 3.** Total educational debt ratio at time of graduation: first-generation U.S. medical students compared to non-first-generation U.S. medical students **eFigure 4.** Percentage of graduating medical students by A) first-generation status, and by B) parental income **eTable 1.** Parent income quintiles breakdown by U.S. census household income quintiles, 2002-2015 **eTable 2.** Total educational debt at time of graduation quartiles breakdown, 2002-2015 **eTable 3.** Demographic data of matriculating first-year U.S. medical students, 2002-2015 **eTable 4.** Comparison of proportions of first-generation and non-first-generation U.S. medical students with parental income in different quintiles of US household income **eTable 5.** Residency specialties of graduating U.S. medical students **eTable 6.** Comparison of socioeconomic composition of U.S. households, U.S. Non-first-generation medical students, and US First generation medical students by race and ethnicity **eTable 7.** Proportion of medical student cohort graduating versus not from medical school, 2002-2012 **eTable 8.** Attrition from medical school among US medical matriculants 2002-2012

Supplement 2.
Data Sharing Statement
