# Supplementary material for: First-Generation and Low-Income Students in the National Medical Student Body
Source: JAMA Netw Open. 2025 May 12;8(5):e259769. doi: 10.1001/jamanetworkopen.2025.9769 (PMC12070234; doi:10.1001/jamanetworkopen.2025.9769)
Supplement: Supplement 1. — eFigure 1. Parental income of first-generation U.S. medical students eFigure 2. Ratio of median first-generation U.S. medical student parental income to non-first-generation U.S. medical student parental income, expressed as percent per year eFigure 3. Total educational debt ratio at time of graduation: first-generation U.S. medical students compared to non-first-generation U.S. medical students eFigure 4. Percentage of graduating medical students by A) first-generation status, and by B) parental income eTable 1. Parent income quintiles breakdown by U.S. census household income quintiles, 2002-2015 eTable 2. Total educational debt at time of graduation quartiles breakdown, 2002-2015 eTable 3. Demographic data of matriculating first-year U.S. medical students, 2002-2015 eTable 4. Comparison of proportions of first-generation and non-first-generation U.S. medical students with parental income in different quintiles of US household income eTable 5. Residency specialties of graduating U.S. medical students eTable 6. Comparison of socioeconomic composition of U.S. households, U.S. Non-first-generation medical students, and US First generation medical students by race and ethnicity eTable 7. Proportion of medical student cohort graduating versus not from medical school, 2002-2012 eTable 8. Attrition from medical school among US medical matriculants 2002-2012 [file jamanetwopen-e259769-s001.pdf]

Supplementary Online Content

Kamran SC, Pompa IR, Nguyen HB, et al. First-generation and low-income students in the national medical student body. *JAMA Netw Open*. 2025;8(5):e259769. doi:10.1001/jamanetworkopen.2025.9769

eFigure 1. Parental income of first-generation U.S. medical students
eFigure 2. Ratio of median first-generation U.S. medical student parental income to non-first-generation U.S. medical student parental income, expressed as percent per year
eFigure 3. Total educational debt ratio at time of graduation: first-generation US medical students compared to non-first-generation US medical students
eFigure 4. Percentage of graduating medical students by A) first-generation status, and by B) parental income
eTable 1. Parent income quintiles breakdown by US census household income quintiles, 2002-2015
eTable 2. Total educational debt at time of graduation quartiles breakdown, 2002-2015
eTable 3. Demographic data of matriculating first-year US medical students, 2002-2015
eTable 4. Comparison of proportions of first-generation and non-first-generation US medical students with parental income in different quintiles of US household income
eTable 5. Residency specialties of graduating US medical students
eTable 6. Comparison of socioeconomic composition of U.S. households, U.S. Non-first-generation medical students, and U.S. First generation medical students by race and ethnicity
eTable 7. Proportion of medical student cohort graduating versus not from medical school, 2002-2012
eTable 8. Attrition from medical school among US medical matriculants 2002-2012

This supplementary material has been provided by the authors to give readers additional information about their work.

© 2025 Kamran SC et al. *JAMA Network Open*.

eFigures

eFigure 1. Parental income of first-generation U.S. medical students. A) Percentage of first-generation U.S. medical students in lowest quartile of parental income per year B) Excess percentage of first-generation U.S. medical students in lowest quartile of total parental income per year

A)

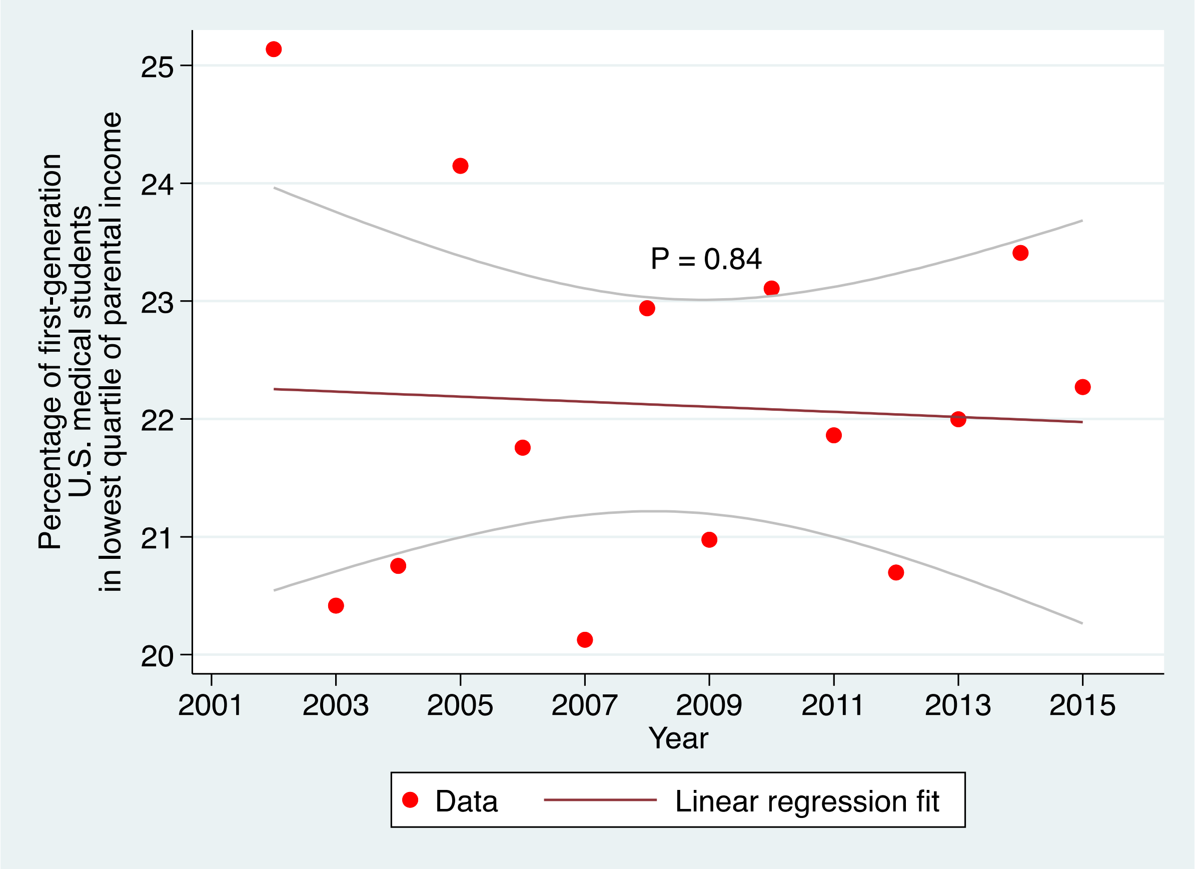

© 2025 Kamran SC et al. *JAMA Network Open*.

B)

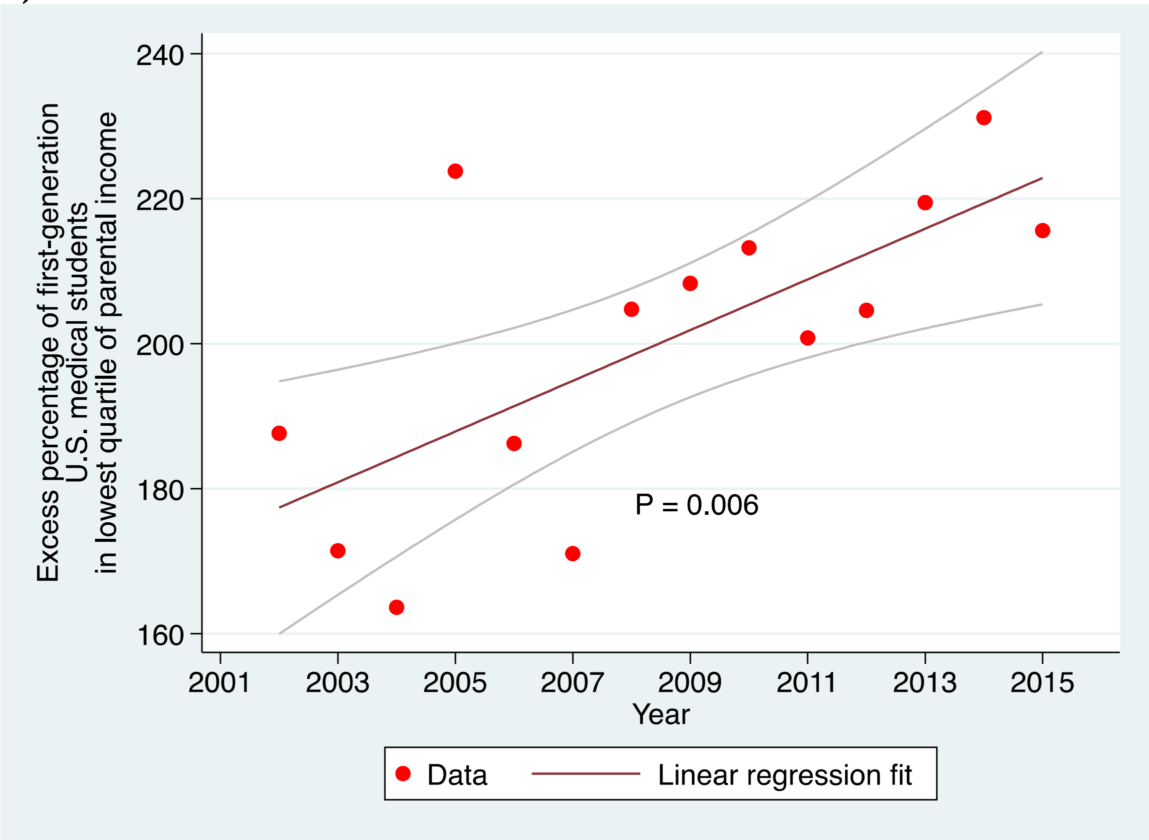

© 2025 Kamran SC et al. *JAMA Network Open*.

eFigure 2. **Ratio of median first-generation U.S. medical student parental income to non-first-generation U.S. medical student parental income, expressed as percent per year**

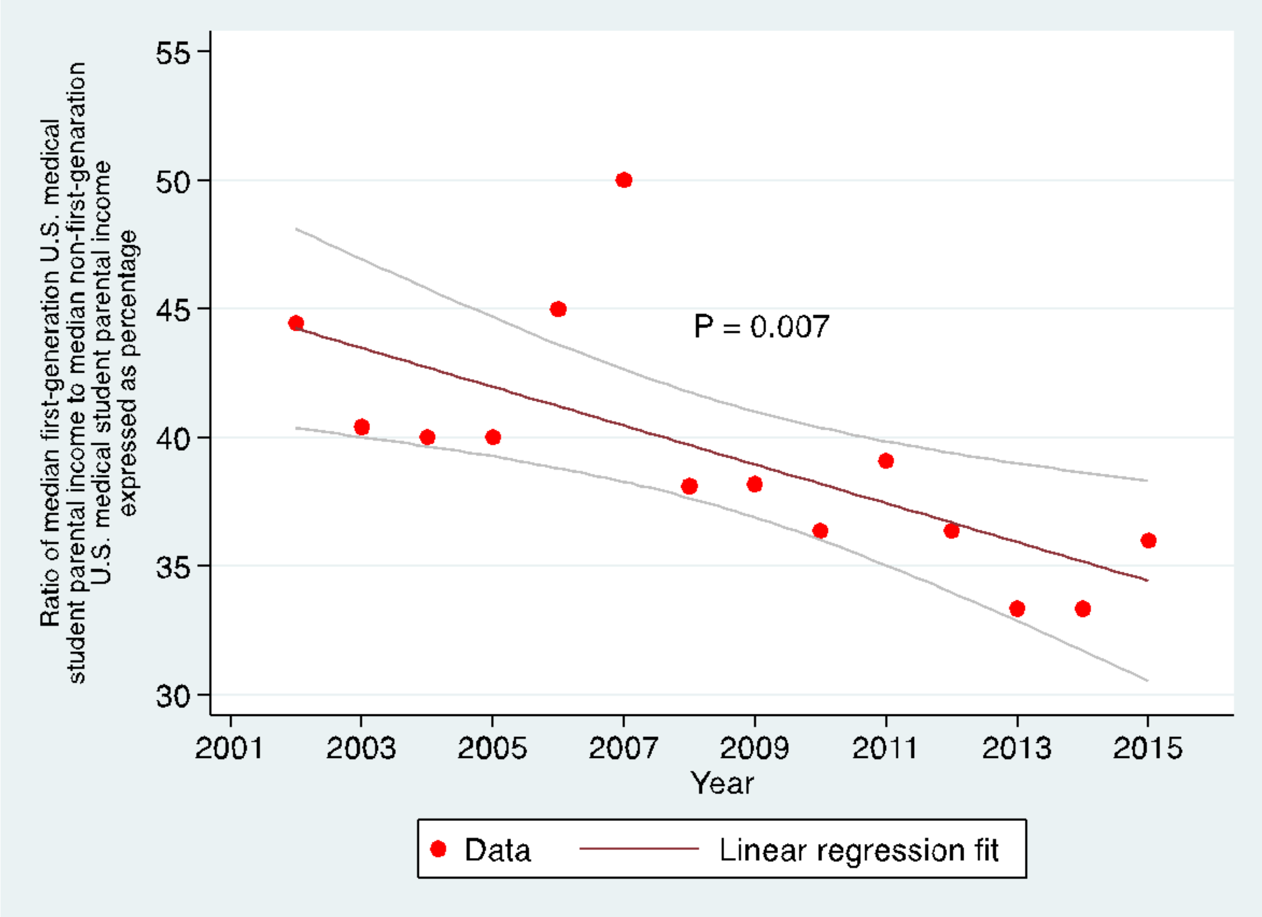

© 2025 Kamran SC et al. *JAMA Network Open*.

eFigure 3. Total educational debt ratio at time of graduation: first-generation US medical students compared to non-first-generation US medical students

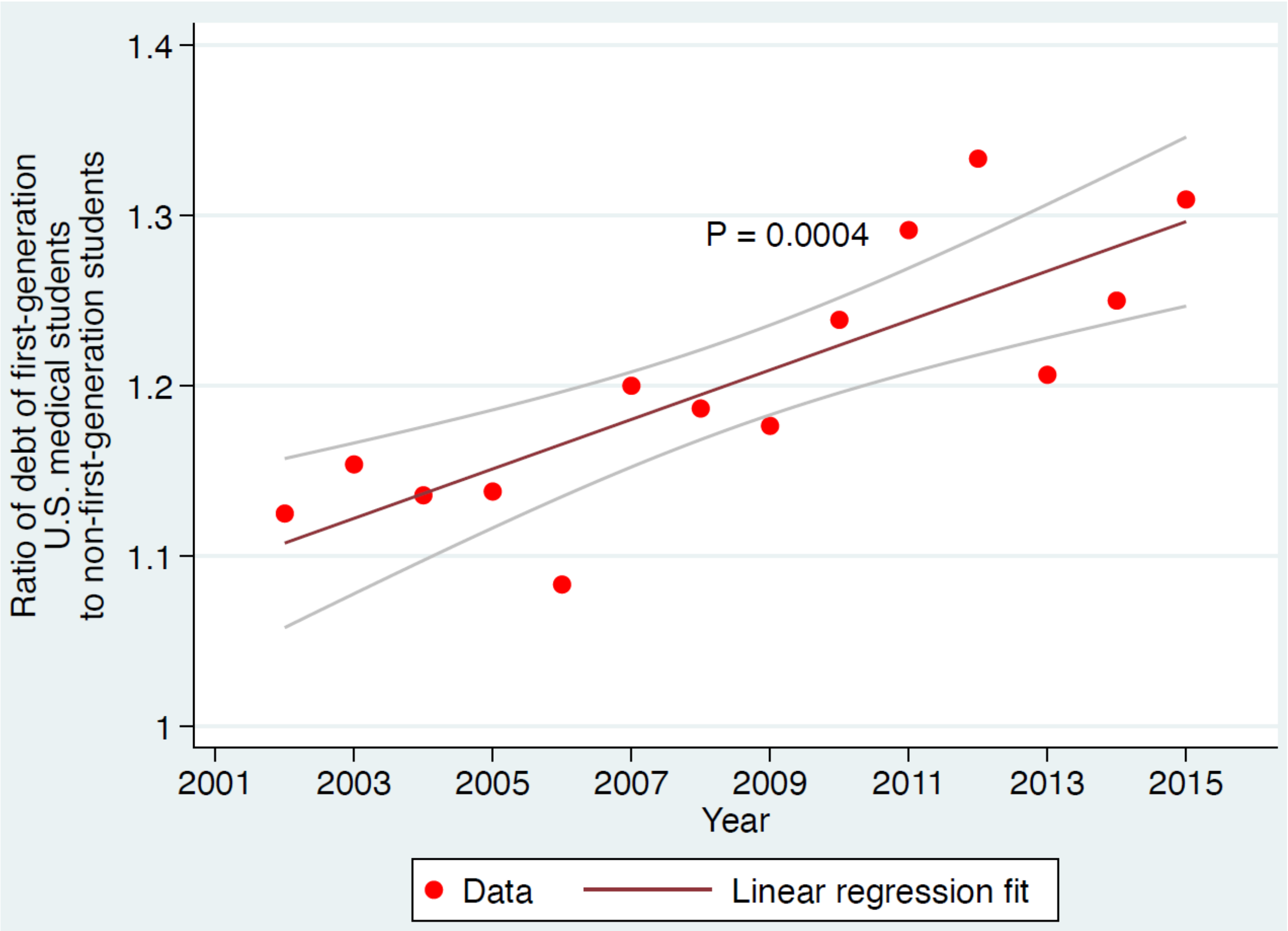

© 2025 Kamran SC et al. *JAMA Network Open*.

eFigure 4. **Percentage of graduating medical students by A) first-generation status, and by B) parental income**

A)

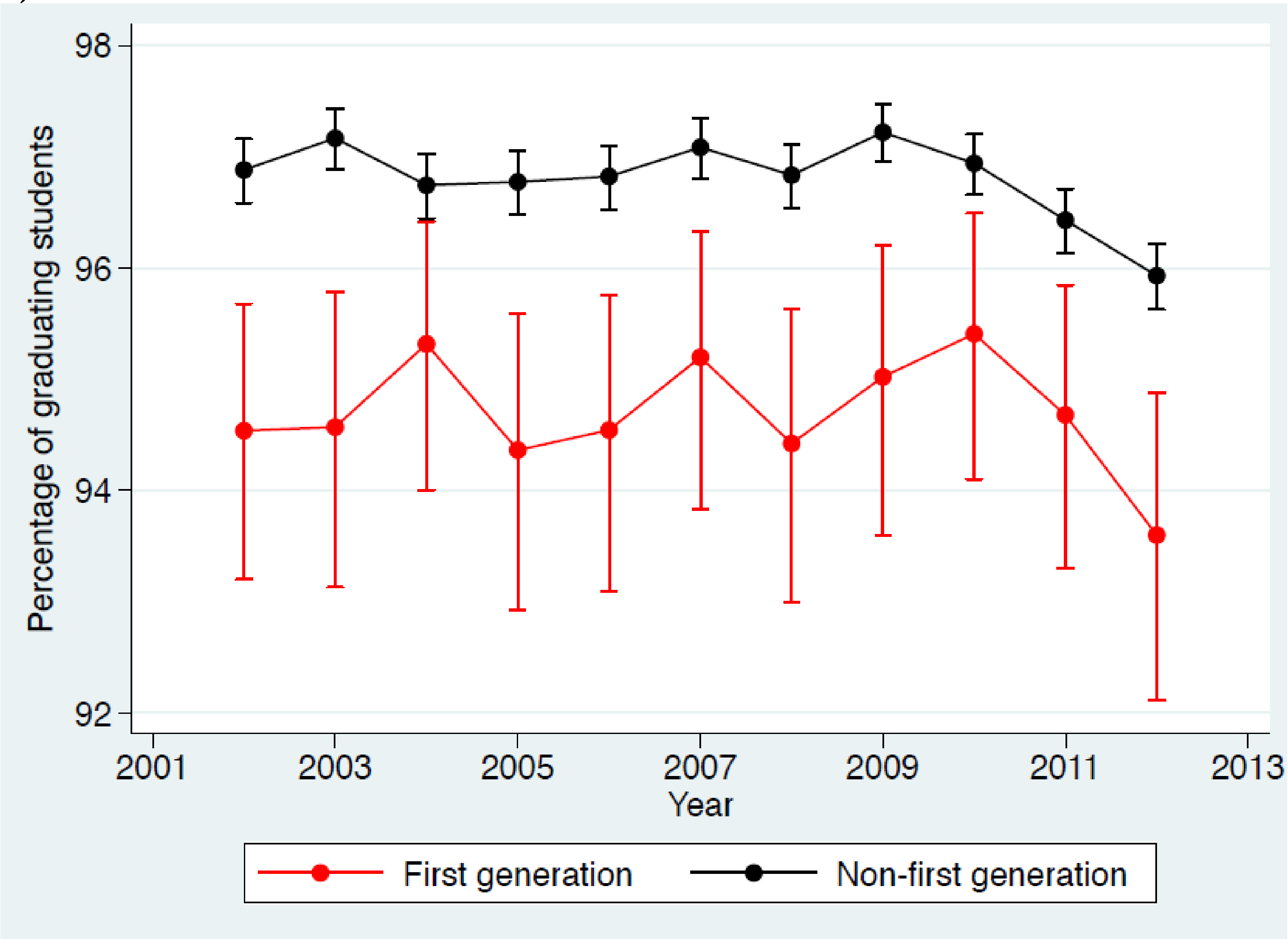

© 2025 Kamran SC et al. *JAMA Network Open*.

B)

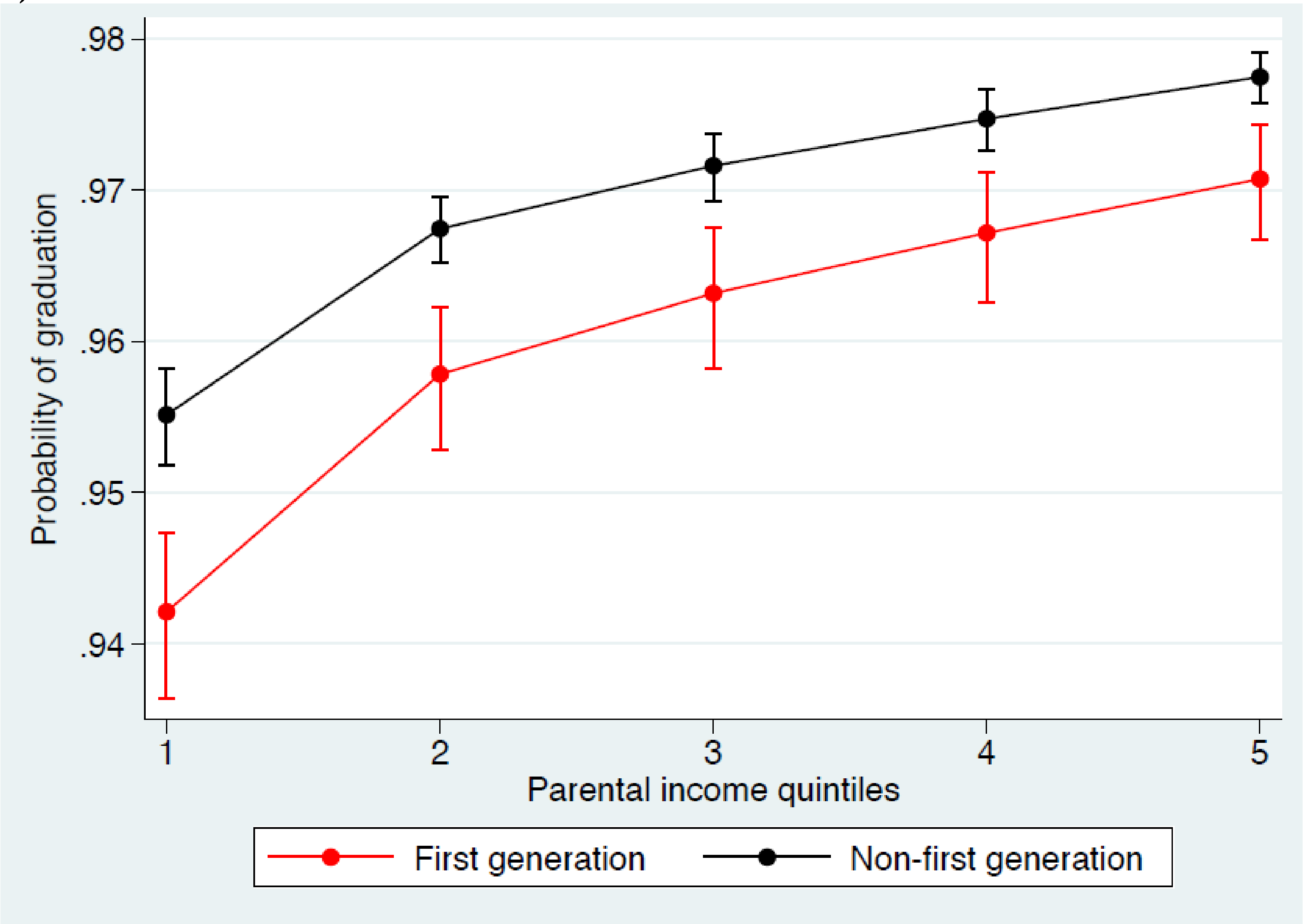

© 2025 Kamran SC et al. *JAMA Network Open*.

eTables

eTable 1. Parent income quintiles breakdown by US census household income quintiles, 2002-2015

	**2002**	**2003**	**2004**	**2005**	**2006**	**2007**	**2008**	**2009**	**2010**	**2011**	**2012**	**2013**	**2014**	**2015**
Quintile 1	$0-$17,970	$0-$17,916	$0-$17,984	$0-$18,486	$0-$19,178	$0-$20,035	$0-$20,291	$0-$20,712	$0-$20,453	$0-$20,000	$0-$20,262	$0-$20,599	$0-$21,000	$0-$21,432
Quintile 2	$17,971-$33,314	$17,917-$33,377	$17,985-$34,000	$18,487-$34,675	$19,179-$36,000	$20,036-$37,774	$20,292-$39,100	$20,713-$39,000	$20,454-$38,550	$20,001-$38,000	$20,263-$38,520	$20,600-$39,764	$21,001-$41,035	$21.433-$41,186
Quintile 3	$33,315-$53,000	$33,378-$53,162	$34,001-$54,453	$34,676-$55,230	$36,001-$57,660	$37,775-$60,000	$39,101-$62,000	$39,001-$62,725	$38,551-$61,801	$38,001-$61,500	$38,521-$62,434	$39,765-$64,582	$41,036-$67,200	$41,187-$68,212
Quintile 4	$53,001-$83,500	$53,163-$84,016	$54,454-$86,867	$55,231-$88,002	$57,661-$91,705	$60,001-$97,032	$62,001-$100,000	$62,726-$100,240	$61,802-$100,000	$61,501-$100,029	$62,435-$101,582	$64,583-$104,096	$67,201-$110,232	$68,213-$112,262
80%-95%	$83,501-$150,499	$84,017-$150,001	$86,868-$154,119	$88,003-$157,151	$91,706-$165,999	$97,033-$174,011	$100,001-$176,999	$100,241-$179,999	$100,001-$180,000	$100,030-$180,484	$101,583-$185,999	$104,097-$191,155	$110,233-$205,127	$112,263-$206,567
Top 5%	>$150,499	>$150,002	>$154,120	>$157,152	>$160,000	>$174,012	>$177,000	>$180,000	>$180,001	>$180,485	>$186,000	>191,156	>$205,128	>206,568

**US Household Income Quintiles, Tax Policy Center*, <https://www.taxpolicycenter.org/statistics/household-income-quintiles>

© 2025 Kamran SC et al. *JAMA Network Open*.

eTable 2. Total educational debt at time of graduation quartiles breakdown, 2002-2015

	2002	2003	2004	2005	2006	2007	2008	2009	2010	2011	2012	2013	2014	2015
**Minimum**	$0.00	$0.00	$0.00	$0.00	$0.00	$0.00	$0.00	$0.00	$0.00	$0.00	$0.00	$0.00	$0.00	$0.00
**Quartile 1**	$53,000.00	$67,676.75	$68,000.00	$60,000.00	$54,000.00	$0.00	$51,000.00	$50,000.00	$37,000.00	$20,000.00	$5,000.00	$12,000.00	$5,000.00	$0.00
**Quartile 2**	$120,000.00	$133,000.00	$140,000.00	$146,000.00	$150,000.00	$152,000.00	$153,000.00	$160,000.00	$160,000.00	$160,000.00	$153,000.00	$159,800.00	$160,000.00	$160,000.00
**Quartile 3**	$163,500.00	$180,000.00	$190,000.00	$200,000.00	$200,000.00	$220,000.00	$210,000.00	$215,000.00	$225,000.00	$230,000.00	$230,000.00	$237,000.00	$245,000.00	$250,000.00
**Maximum**	$500,000.00	$575,000.00	$600,000.00	$600,000.00	$599,999.00	$613,000.00	$700,000.00	$650,000.00	$640,000.00	$960,000.00	$1,000,000.00	$1,000,000.00	$900,000.00	$778,000.00

© 2025 Kamran SC et al. *JAMA Network Open*.

eTable 3. Demographic data of matriculating first-year US medical students, 2002-2015

	2002	2003	2004	2005	2006	2007	2008	2009	2010	2011	2012	2013	2014	2015	Total
	No. (%)	No. (%)	No. (%)	No. (%)	No. (%)	No. (%)	No. (%)	No. (%)	No. (%)	No. (%)	No. (%)	No. (%)	No. (%)	No. (%)	No. (%)
**Total U.S. Medical Students**	16473	16527	16633	16983	17351	17747	18024	18379	18654	19211	19511	20049	20340	20631	256513
**Gender**															
**Male**	8367(50.8)	8320(50.3)	8402(50.5)	8749(51.5)	8918(51.4)	9173(51.7)	9418(52.3)	9569(52.1)	9901(53.1)	10180(53)	10451(53.6)	10584(52.8)	10623(52.2)	10766(52.2)	133421(52)
**Female**	8106(49.2)	8207(49.7)	8231(49.5)	8234(48.5)	8433(48.6)	8574(48.3)	8606(47.7)	8810(47.9)	8753(46.9)	9031(47)	9060(46.4)	9465(47.2)	9717(47.8)	9861(47.8)	123088(48)
**Unknown**	0(0)	0(0)	0(0)	0(0)	0(0)	0(0)	0(0)	0(0)	0(0)	0(0)	0(0)	0(0)	0(0)	4(0)	4(0)
**Race/Ethnicity**															
Non-Hispanic White	9970(60.5)	10119(61.2)	10335(62.1)	10329(60.8)	10592(61)	10662(60.1)	10635(59)	10692(58.2)	10655(57.1)	11058(57.6)	11009(56.4)	10372(51.7)	10608(52.2)	10571(51.2)	147607(57.5)
Non-Hispanic Black	1122(6.8)	1058(6.4)	1086(6.5)	1071(6.3)	1158(6.7)	1146(6.5)	1142(6.3)	1136(6.2)	1171(6.3)	1182(6.2)	1181(6.1)	1234(6.2)	1227(6)	1349(6.5)	16263(6.3)
Non-Hispanic Asian	3028(18.4)	3040(18.4)	3092(18.6)	3341(19.7)	3283(18.9)	3577(20.2)	3575(19.8)	3731(20.3)	3812(20.4)	3857(20.1)	4068(20.8)	3713(18.5)	3817(18.8)	4095(19.8)	50029(19.5)
Non-Hispanic AIAN/NPI	60(0.4)	40(0.2)	59(0.4)	51(0.3)	100(0.6)	90(0.5)	86(0.5)	77(0.4)	75(0.4)	58(0.3)	77(0.4)	72(0.4)	80(0.4)	72(0.3)	997(0.4)
Non-Hispanic Multiracial	573(3.5)	633(3.8)	504(3)	538(3.2)	456(2.6)	451(2.5)	452(2.5)	501(2.7)	517(2.8)	591(3.1)	617(3.2)	667(3.3)	777(3.8)	792(3.8)	8069(3.1)
Hispanic	1118(6.8)	1079(6.5)	1165(7)	1266(7.5)	1283(7.4)	1275(7.2)	1399(7.8)	1401(7.6)	1530(8.2)	1611(8.4)	1722(8.8)	1822(9.1)	1858(9.1)	1988(9.6)	20517(8)
Other/Unknown	602(3.7)	558(3.4)	392(2.4)	387(2.3)	479(2.8)	546(3.1)	735(4.1)	841(4.6)	894(4.8)	854(4.4)	837(4.3)	2169(10.8)	1973(9.7)	1764(8.6)	13031(5.1)
**URiM**															
Yes	2300(14)	2177(13.2)	2310(13.9)	2388(14.1)	2541(14.6)	2511(14.1)	2627(14.6)	2614(14.2)	2776(14.9)	2851(14.8)	2980(15.3)	3128(15.6)	3165(15.6)	3409(16.5)	37777(14.7)
No	13964(84.8)	14138(85.5)	14103(84.8)	14332(84.4)	14531(83.7)	14910(84)	15107(83.8)	15525(84.5)	15598(83.6)	16132(84)	16265(83.4)	15274(76.2)	15725(77.3)	15955(77.3)	211559(82.5)
Unknown	209(1.3)	212(1.3)	220(1.3)	263(1.5)	279(1.6)	326(1.8)	290(1.6)	240(1.3)	280(1.5)	228(1.2)	266(1.4)	1647(8.2)	1450(7.1)	1267(6.1)	7177(2.8)
**First Generation**															
Yes	1373(8.3)	1197(7.2)	1260(7.6)	1224(7.2)	1191(6.9)	1208(6.8)	1237(6.9)	1145(6.2)	1263(6.8)	1278(6.7)	1281(6.6)	1342(6.7)	1394(6.9)	1414(6.9)	17807(6.9)
No	14338(87)	14718(89.1)	14747(88.7)	15189(89.4)	14479(83.4)	15061(84.9)	15197(84.3)	15686(85.3)	15858(85)	16307(84.9)	17572(90.1)	18148(90.5)	18328(90.1)	18624(90.3)	224252(87.4)
Unknown	762(4.6)	612(3.7)	626(3.8)	570(3.4)	1681(9.7)	1478(8.3)	1590(8.8)	1548(8.4)	1533(8.2)	1626(8.5)	658(3.4)	559(2.8)	618(3)	593(2.9)	14454(5.6)

© 2025 Kamran SC et al. *JAMA Network Open*.

**Low-Income**															
Yes	1320(8)	1214(7.3)	1096(6.6)	989(5.8)	1078(6.2)	1089(6.1)	1194(6.6)	1235(6.7)	1352(7.2)	1139(5.9)	1245(6.4)	1286(6.4)	1474(7.2)	1267(6.1)	16978(6.6)
No	10375(63)	10129(61.3)	9246(55.6)	8685(51.1)	9425(54.3)	10014(56.4)	10985(60.9)	10643(57.9)	10645(57.1)	9758(50.8)	10106(51.8)	10881(54.3)	10613(52.2)	9644(46.7)	141149(55)
Unknown	4778(29)	5184(31.4)	6291(37.8)	7309(43)	6848(39.5)	6644(37.4)	5845(32.4)	6501(35.4)	6657(35.7)	8314(43.3)	8160(41.8)	7882(39.3)	8253(40.6)	9720(47.1)	98386(38.4)
**Total Educational Debt at Graduation**															
Quartile 1	2110(12.8)	2189(13.2)	2753(16.6)	2641(15.6)	2958(17)	1775(10)	3028(16.8)	3258(17.7)	3214(17.2)	3398(17.7)	3368(17.3)	3432(17.1)	3597(17.7)	3707(18)	41428(16.2)
Quartile 2	2231(13.5)	2197(13.3)	2782(16.7)	2511(14.8)	3259(18.8)	1664(9.4)	3028(16.8)	3332(18.1)	3481(18.7)	3485(18.1)	3333(17.1)	3427(17.1)	3657(18)	3596(17.4)	41983(16.4)
Quartile 3	1967(11.9)	2379(14.4)	2784(16.7)	3049(18)	2928(16.9)	1733(9.8)	3068(17)	2859(15.6)	2999(16.1)	3200(16.7)	3382(17.3)	3431(17.1)	3524(17.3)	3883(18.8)	41186(16.1)
Quartile 4	2102(12.8)	1989(12)	2678(16.1)	2102(12.4)	2674(15.4)	1698(9.6)	2962(16.4)	3122(17)	3140(16.8)	3230(16.8)	3310(17)	3427(17.1)	3563(17.5)	3196(15.5)	39193(15.3)
Unknown	8063(48.9)	7773(47)	5636(33.9)	6680(39.3)	5532(31.9)	10877(61.3)	5938(32.9)	5808(31.6)	5820(31.2)	5898(30.7)	6118(31.4)	6332(31.6)	5999(29.5)	6249(30.3)	92723(36.1)
**Total Educational Debt at Graduation (median)**															
First-generation	$135,000	$150,000	$159,000	$165,000	$162,500	$180,000	$178,000	$185,000	$192,000	$195,000	$200,000	$187,000	$200,000	$209,500	-
Non-first-generation	$120,000	$130,000	$140,000	$145,000	$150,000	$150,000	$150,000	$157,250	$155,000	$151,000	$150,000	$155,000	$160,000	$160,000	-
Ratio	1.125	1.154	1.136	1.138	1.083	1.200	1.187	1.176	1.239	1.291	1.333	1.206	1.250	1.309	-
**Median Parental Income**															
First-generation	$40,000	$40,000	$40,000	$40,000	$45,000	$50,000	$40,000	$42,000	$40,000	$43,000	$40,000	$40,000	$40,000	$45,000	-
Non-first-generation	$90,000	$99,000	$100,000	$100,000	$100,000	$100,000	$105,000	$110,000	$110,000	$110,000	$110,000	$120,0000	$120,000	$125,000	-
**First Generation US Medical Students in Lowest quartile Of parental income (%)**	25	20.4	20.8	24.1	21.8	20.1	22.9	21.0	23.1	21.9	20.7	22.0	23.4	22.3	-

© 2025 Kamran SC et al. *JAMA Network Open*.

**Excess %'age of first generation US medical students in lowest quartile of parental income (%)**	187.7	171.4	163.6	223.8	186.2	171.1	204.8	208.3	213.2	200.8	204.6	219.5	231.2	215.6	-

eTable 4. Comparison of proportions of first-generation and non-first-generation US medical students with parental income in different quintiles of US household income

	**Top 5%**			**80-95%**			**4th Quintile**		
**Year**	**All Students**	**Non-First-Generation Students**	**First-Generation Students**	**All students**	**Non-First-Generation Students**	**First-Generation Students**	**All students**	**Non-First-Generation Students**	**First-Generation Students**
2002	19.6%	21.1%	1.4%	34.0%	35.9%	9.1%	20.8%	20.6%	23.2%
2003	21.1%	22.4%	1.7%	34.6%	36.2%	11.8%	20.9%	20.7%	23.8%
2004	22.7%	24.1%	2.8%	33.8%	35.3%	12.7%	20.4%	20.2%	23.1%
2005	25.9%	27.6%	2.6%	33.2%	34.9%	10.1%	18.6%	18.5%	19.9%
2006	26.1%	27.4%	4.4%	31.2%	32.6%	10.3%	21.7%	21.5%	27.5%
2007	28.1%	29.7%	3.8%	31.7%	33.1%	10.1%	16.7%	16.4%	21.3%
2008	29.7%	31.5%	2.8%	20.1%	21.1%	6.5%	27.9%	28.1%	23.2%
2009	28.6%	30.5%	2.1%	21.9%	22.9%	6.1%	26.7%	26.7%	25.2%
2010	28.7%	31.3%	2.3%	21.8%	23.3%	5.9%	25.7%	25.9%	21.7%
2011	30.3%	32.9%	1.9%	21.8%	23.1%	2.4%	25.2%	25.1%	24.2%
2012	30.3%	32.6%	2.6%	22.1%	23.3%	2.8%	24.9%	25.1%	22.4%
2013	37.1%	34.6%	3.0%	26.2%	24.0%	2.1%	26.5%	22.9%	22.2%
2014	23.4%	25.2%	1.2%	30.7%	32.7%	6.3%	23.6%	24.0%	18.8%
2015	24.5%	26.4%	0.8%	31.0%	33.0%	6.2%	23.0%	23.0%	23.1%
	**3rd Quintile**			**2nd Quintile**			**Bottom 1st quintile**		
**Year**	**All Students**	**Non-First-Generation Students**	**First-Generation Students**	**All students**	**Non-First-Generation Students**	**First-Generation Students**	**All students**	**Non-First-Generation Students**	**First-Generation Students**
2002	14.3%	13.1%	28.8%	7.8%	6.5%	24.2%	3.5%	2.7%	13.3%
2003	12.7%	11.8%	26.2%	7.4%	6.2%	24.3%	3.3%	2.7%	12.1%
2004	12.5%	11.5%	26.5%	7.1%	6.1%	20.6%	3.5%	2.8%	14.3%

© 2025 Kamran SC et al. *JAMA Network Open*.

2005	12.1%	10.9%	27.7%	6.5%	5.4%	22.8%	3.7%	2.8%	16.8%
2006	10.7%	10.0%	21.4%	6.6%	5.6%	22.9%	3.6%	2.9%	13.6%
2007	13.6%	12.7%	29.4%	5.4%	4.7%	17.2%	4.4%	3.4%	18.3%
2008	12.5%	11.5%	26.6%	6.4%	4.4%	20.2%	4.4%	3.3%	20.7%
2009	12.5%	11.4%	27.7%	6.7%	4.7%	19.6%	4.7%	3.7%	19.3%
2010	12.6%	11.2%	26.9%	6.4%	5.0%	22.0%	4.8%	3.3%	21.1%
2011	12.3%	10.8%	28.8%	5.5%	4.3%	19.9%	5.0%	3.7%	19.8%
2012	11.8%	10.7%	25.4%	6.2%	4.9%	22.4%	4.8%	3.5%	21.4%
2013	13.5%	10.5%	25.7%	6.8%	4.6%	22.4%	5.4%	3.3%	21.6%
2014	10.1%	9.0%	22.9%	7.6%	5.8%	29.2%	4.6%	3.3%	21.6%
2015	9.8%	8.7%	24.2%	7.5%	5.8%	28.6%	4.1%	3.1%	17.1%

© 2025 Kamran SC et al. *JAMA Network Open*.

eTable 5. Residency specialties of graduating US medical students

	All U.S. Medical Students	Non-First Generation Students	First Generation Students	Unknown Generation Status Students
**Emergency Medicine**	19322(7.5)	16861(7.5)	1407(7.9)	1054(7.3)
**Psychiatry**	11187(4.4)	9679(4.3)	723(4.1)	785(5.4)
**Internal Medicine**	66294(25.8)	58338(26)	4397(24.7)	3559(24.6)
**Anesthesiology**	9814(3.8)	8342(3.7)	795(4.5)	677(4.7)
**Transitional Year**	12044(4.7)	10756(4.8)	692(3.9)	596(4.1)
**Family Medicine**	21869(8.5)	18268(8.1)	2099(11.8)	1502(10.4)
**Pediatrics**	30444(11.9)	27220(12.1)	1720(9.7)	1504(10.4)
**Surgery**	35851(14)	31691(14.1)	2274(12.8)	1886(13)
**Preventive Medicine**	77(0)	67(0)	7(0)	3(0)
**Pathology-Anatomic and Clinical**	3846(1.5)	3279(1.5)	325(1.8)	242(1.7)
**Neurology**	2308(0.9)	2036(0.9)	146(0.8)	126(0.9)
**Hospice and Palliative Medicine**	11(0)	11(0)	0(0)	0(0)
**Radiology-Diagnostic**	1129(0.4)	966(0.4)	75(0.4)	88(0.6)
**Otolaryngology**	3857(1.5)	3530(1.6)	167(0.9)	160(1.1)
**Allergy and Immunology**	25(0)	21(0)	3(0)	1(0)
**Urology**	899(0.4)	799(0.4)	57(0.3)	43(0.3)
**Obstetrics and Gynecology**	13409(5.2)	11766(5.2)	907(5.1)	736(5.1)
**Dermatology**	262(0.1)	227(0.1)	11(0.1)	24(0.2)
**Radiation Oncology**	124(0.05)	109(0)	6(0)	9(0.1)
**Plastic Surgery**	1420(0.6)	1298(0.6)	59(0.3)	63(0.4)
**Ophthalmology**	168(0.1)	157(0.1)	3(0)	8(0.1)
**Medical Genetics and Genomics**	4(0)	4(0)	0(0)	0(0)
**Unknown**	22149(8.6)	18827(8.4)	1934(10.9)	1388(9.6)
**Total**	256513	224252	17807	14454

© 2025 Kamran SC et al. *JAMA Network Open*.

eTable 6. Comparison of socioeconomic composition of U.S. households, U.S. Non-first-generation medical students, and U.S. First generation medical students by race and ethnicity

A. 2002

	All respondents			Non-Hispanic Asian			Non-Hispanic Black			Hispanic (any race)			Non-Hispanic White		
	**All US house holds (%)**	**Non 1st Gen (%)**	**1st Gen (%)**	**All US house holds (%)**	**Non 1st Gen (%)**	**1st Gen (%)**	**All US househ olds (%)**	**Non 1st Gen (%)**	**1st Gen (%)**	**All US househ olds (%)**	**Non 1st Gen (%)**	**1st Gen (%)**	**All US househ olds (%)**	**Non 1st Gen (%)**	**1st Gen (%)**
1st Quintile: $0-$17,970	20.0	2.7	13.3	15.2	3.2	21.7	32.4	5.7	16.9	24.2	7.5	27.3	17.5	1.5	7.4
2nd Quintile: $17,971-$33,314	20.0	6.5	24.2	16.5	7.6	31.0	24.0	12.6	32.3	26.1	13.8	25.0	18.7	4.5	19.4
3rd Quintile: $33,315-$53,000	20.0	13.1	28.8	18.8	11.6	24.8	19.2	21.3	23.1	21.7	15.7	26.5	19.9	12.2	31.2
4th Quintile: $53,001-$83,500	20.0	20.6	23.2	20.7	19.6	12.4	14.6	23.1	23.1	17.1	21.4	17.4	21.3	20.6	28.4
80%-90%: $83,501-$150,499	15.0	36.0	9.1	20.6	36.0	9.3	7.9	27.2	4.6	8.8	27.2	3.8	16.8	38.1	11.4
Top 5%: >$150,499	5.0	21.1	1.4	8.1	21.9	0.8	1.9	10.2	0.0	2.2	14.5	0.0	5.8	23.0	2.2

© 2025 Kamran SC et al. *JAMA Network Open*.

B. 2008

	**All respondents**			**Non-Hispanic Asian**			**Non-Hispanic Black**			**Hispanic (any race)**			**Non-Hispanic White**		
	**All US households (%)**	**Non 1st Gen (%)**	**1st Gen (%)**	**All US households (%)**	**Non 1st Gen (%)**	**1st Gen (%)**	**All US households (%)**	**Non 1st Gen (%)**	**1st Gen (%)**	**All US households (%)**	**Non 1st Gen (%)**	**1st Gen (%)**	**All US households (%)**	**Non 1st Gen (%)**	**1st Gen (%)**
1st Quintile: $0-$20,291	20.0	3.3	20.7	16.5	4.2	26.8	31.7	10.1	42.3	26.0	5.9	22.7	17.1	2.1	7.8
2nd Quintile: $20,292-$39,100	20.0	4.4	20.2	15.1	5.1	25.0	24.4	8.9	19.2	25.1	10.7	31.8	18.7	3.0	12.0
3rd Quintile: $39,101-$62,000	20.0	11.5	26.6	17.1	11.3	28.6	19.9	20.6	23.1	21.6	16.9	21.2	19.9	9.9	30.4
4th Quintile: $62,001-$100,000	20.0	28.1	23.2	20.1	30.1	13.7	14.3	30.3	12.8	16.0	28.5	17.4	21.6	27.5	35.0
80%-90%: $100,001-$176,999	15.0	21.1	6.5	22.3	19.2	2.4	8.1	15.6	2.6	9.4	16.7	4.5	16.8	22.8	11.0
Top 5%: >$177,000	5.0	31.5	2.8	8.9	30.1	3.6	1.5	14.5	0.0	1.9	21.3	2.3	5.9	34.8	3.9

© 2025 Kamran SC et al. *JAMA Network Open*.

C. 2015

	**All respondents**			**Non-Hispanic Asian**			**Non-Hispanic Black**			**Hispanic (any race)**			**Non-Hispanic White**		
	**All US households (%)**	**Non 1st Gen (%)**	**1st Gen (%)**	**All US households (%)**	**Non 1st Gen (%)**	**1st Gen (%)**	**All US households (%)**	**Non 1st Gen (%)**	**1st Gen (%)**	**All US households (%)**	**Non 1st Gen (%)**	**1st Gen (%)**	**All US households (%)**	**Non 1st Gen (%)**	**1st Gen (%)**
1st Quintile: $0-$21,432	19.9	3.1	17.1	15.2	4.0	18.8	33.2	6.6	31.7	24.0	6.3	22.8	16.8	1.6	7.1
2nd Quintile: $28,084-$41,186	20.0	5.8	28.6	13.6	5.6	38.1	23.2	14.1	30.5	24.9	12.3	27.5	18.9	4.0	19.2
3rd Quintile: $41,186-$68,212	20.0	8.7	24.2	18.1	8.6	21.5	19.6	17.4	22.0	22.2	12.3	22.8	19.8	7.3	27.9
4th Quintile: $68,213-$112,262	20.0	23.0	23.1	21.4	23.3	18.8	14.2	26.2	14.6	16.8	26.6	19.9	21.7	21.8	33.3
80%-95%: $112,263-$206,567	15.0	33.0	6.2	21.6	34.9	1.7	7.9	23.8	1.2	9.6	24.5	5.8	17.0	35.1	11.7
Top 5%: => 206,568	5.0	26.5	0.8	10.1	23.5	1.1	1.9	11.9	0.0	2.6	17.8	1.2	5.7	30.2	0.8

© 2025 Kamran SC et al. *JAMA Network Open*.

eTable 7. Proportion of medical student cohort graduating versus not from medical school, 2002-2012

Graduating Students																						
	2002		2003		2004		2005		2006		2007		2008		2009		2010		2011		2012	
	N	%	N	%	N	%	N	%	N	%	N	%	N	%	N	%	N	%	N	%	N	%
**All US. Medical Students**	15904	96.5	16015	96.9	16065	96.6	16392	96.5	16714	96.3	17175	96.8	17397	96.5	17809	96.9	18020	96.6	18477	96.2	18665	95.7
**Non-First Generation U.S. Medical Students**	13891	96.9	14301	97.2	14267	96.7	14699	96.8	14019	96.8	14622	97.1	14716	96.8	15250	97.2	15373	96.9	15725	96.4	16857	95.9
**First Generation US. Medical Students**	1298	94.5	1132	94.6	1201	95.3	1155	94.4	1126	94.5	1150	95.2	1168	94.4	1088	95	1205	95.4	1210	94.7	1199	93.6
**Unknown Generation Status**	715	93.8	582	95.1	597	95.4	538	94.4	1569	93.3	1403	94.9	1513	95.2	1471	95	1442	94.1	1542	94.8	609	92.6
**Non-Graduating Students**																						
**All US. Medical Students**	569	3.5	512	3.1	568	3.4	591	3.5	637	3.7	572	3.2	627	3.5	570	3.1	634	3.4	734	3.8	846	4.3
**Non-First Generation U.S. Medical Students**	447	3.1	417	2.8	480	3.3	490	3.2	460	3.2	439	2.9	481	3.2	436	2.8	485	3.1	582	3.6	715	4.1
**First Generation US. Medical Students**	75	5.5	65	5.4	59	4.7	69	5.6	65	5.5	58	4.8	69	5.6	57	5	58	4.6	68	5.3	82	6.4
**Unknown Generation Status**	47	6.2	30	4.9	29	4.6	32	5.6	112	6.7	75	5.1	77	4.8	77	5	91	5.9	84	5.2	49	7.4

© 2025 Kamran SC et al. *JAMA Network Open*.

eTable 8. Attrition from medical school among US medical matriculants 2002-2012

**Characteristic**			
	**Total No.**	**Attrition No. (%)**	**Graduated No. (%)**
**All**	195493	6860	188633
**Race and ethnicity**			
Hispanic	14849 (7.6)	887 (12.9)	13962 (7.4)
**Non-Hispanic**			
AIAN/NPI	773 (0.4)	58 (0.9)	715 (0.5)
Asian	38404 (19.6)	1175 (17.2)	37229 (19.7)
Black/African American	12453 (6.4)	884 (12.9)	11569 (6.1)
Multiracial	5833 (3.0)	243 (3.5)	5590 (3.0)
White	116056 (59.4)	3370 (49.1)	112686 (59.7)
Unknown/Other/Declined	7125 (3.6)	243 (3.5)	6882 (3.6)
**Low-income***			
No	110011 (56.3)	3199 (46.6)	106812 (56.6)
Yes	12951 (6.6)	672 (9.8)	12279 (6.5)
Unknown Parent Income	72531 (37.1)	2989 (43.6)	69542 (36.9)
**First Generation**			
No	169152 (86.5)	5432 (79.2)	163720 (86.8)
Yes	13657 (7.0)	725 (10.6)	12932 (6.8)
Unknown Generation Status	12684 (6.5)	703 (10.2)	11981 (6.4)

*Low-income: parental income fell in bottom two quintiles

© 2025 Kamran SC et al. *JAMA Network Open*.
