# Supplementary material for: First-Generation and Low-Income Students in the National Medical Student Body
Source: JAMA Netw Open. 2025 May 12;8(5):e259769. doi: 10.1001/jamanetworkopen.2025.9769 (PMC12070234; doi:10.1001/jamanetworkopen.2025.9769)
Supplement: Supplement 2. — Data Sharing Statement [file jamanetwopen-e259769-s002.pdf]

Data Sharing Statement

Kamran. First-Generation and Low-Income Students in the National Medical Student Body. *JAMA Netw Open*. Published May 12, 2025. doi:10.1001/jamanetworkopen.2025.9769

Data
Data available: No

Additional Information
Explanation for why data not available: Data for the study can be requested from the AAMC and they have to give approval for release and use of the datasets.
